# Feature selection for high-dimensional temporal data

**DOI:** 10.1186/s12859-018-2023-7

**Published:** 2018-01-23

**Authors:** Michail Tsagris, Vincenzo Lagani, Ioannis Tsamardinos

**Affiliations:** Department of Computer Science, University of Crete, Voutes Campus, Heraklion, 70013 Greece

**Keywords:** Time course data, Longitudinal data, Regression, Variable selection, Multiple solutions

## Abstract

**Background:**

Feature selection is commonly employed for identifying collectively-predictive biomarkers and biosignatures; it facilitates the construction of small statistical models that are easier to verify, visualize, and comprehend while providing insight to the human expert. In this work we extend established constrained-based, feature-selection methods to high-dimensional “omics” temporal data, where the number of measurements is orders of magnitude larger than the sample size. The extension required the development of conditional independence tests for temporal and/or static variables conditioned on a set of temporal variables.

**Results:**

The algorithm is able to return multiple, equivalent solution subsets of variables, scale to tens of thousands of features, and outperform or be on par with existing methods depending on the analysis task specifics.

**Conclusions:**

The use of this algorithm is suggested for variable selection with high-dimensional temporal data.

**Electronic supplementary material:**

The online version of this article (doi:10.1186/s12859-018-2023-7) contains supplementary material, which is available to authorized users.

## Background

Temporal data measure a set of time-varying quantities over time on a population. They are often employed to understand the dynamics of evolution of a system, the effects of a perturbation (interventional studies), or the differences in dynamics between two groups (such as in case-control studies). Such data arise in many fields, namely bioinformatics, medicine, agriculture and econometrics, just to name a few.

Two broad categories of temporal data can be defined, depending on the sampling procedure: *longitudinal* data arise when *the same samples* are repeatedly measured at different times points, while *time–course* (a.k.a repeated cross-sectional) data are produced when *distinct samples* (from the same population) are measured at each time point (e.g., in case of destructive testing). In contrast, *time-series data* that often arise in econometrics, measure samples at regular time intervals and are often of a much larger temporal extent than temporal data in biology.

The correlation structure of temporal data, which includes auto-correlation of the same quantity over time or over the same sample requires special analysis techniques. For example, longitudinal data are often modeled with mixed models, which allow to properly account for within-subject correlations.

Feature selection (a.k.a. variable selection) in predictive modeling can be defined as the task of selecting one or more minimal-size and (collectively) optimally predictive feature subsets for a target outcome. Reducing the number of features results in smaller, easier-to-verify, understand, visualize, and apply predictive models; most importantly perhaps, it provides important insight to the data generating mechanism. This is no accident, as feature selection has been theoretically connected to causal discovery and the causal data generating model [1]. A typical example of a feature selection task is the identification of the genes whose expression allows the early diagnosis of a given disease. In the context of temporal data, each feature has a temporal extent and a time trajectory that can be employed for prediction.

To the best of our knowledge, most variable selection methods proposed so far for temporal data are devised for studies where the number of samples is larger than the number of predictors, i.e., *p*<*n*. This limits the applicability of these algorithms to “omics” types of data such as transcriptomics, epigenomics and genomics, where *p* is usually order of magnitudes larger than *n*.

Constraint-based, Markov-Blanket variable-selection methods form a class of algorithms that are inspired by the theory of (Causal) Bayesian Networks [2] and include HITON, MMMB, MMPC, SES and others [3–5]. The Markov Blanket of the target outcome *T* is defined as a minimal-size set that renders all other variables conditionally independent of *T*. Under certain broad conditions it has been shown to be the solution to the feature selection problem [1]. If the data distribution can be represented with a faithful Bayesian Network (BN) [6] then the Markov Blanket of *T* is unique and has an interesting graphical interpretation: it comprises of the neighbors of *T* (i.e., the parents and children of *T*) and the spouses (parents or common children) of *T* in any such (unknown) faithful BN graph.

*The main contribution of this paper is adapting constraint-based, variable selection methods for temporal data.* Constraint-based methods process the data exclusively through conditional independence tests, repetitively applying these tests for identifying variables that cannot be made independent of *T* conditioned on any other subset, and are thus needed for optimal prediction. As discussed in [7], employing a suitable conditional independence test is sufficient for extending constraint-based methods to new types of data. While such tests exist for various types of data, the idiosyncrasies of temporal data require the development of novel, specific conditional independence tests. We denote with *Ind*(*X*;*T*;**Z**) the test assessing the null hypothesis that *X* is independent of *T* given **Z**. For temporal data some of these variables (but not necessarily all of them) may have temporal extent and be better denoted as *X*_*t*_ instead of *X*, with the index indicating the time-point. The independence test could be implemented as a log likelihood ratio test [8]. The latter fits two nested models, one modeling *T* on **Z** alone and the other on *X*∪**Z**. If the two models are equivalent, then the null hypothesis is not rejected. The modeling strategy used for creating the two nested models depends on the temporal characteristics of the variables involved in the test. However, for linear mixed models, likelihood ratio tests do not have the proper behaviour when the sample size is rather small and hence the use of F tests is suggested [9]. We depict four different scenarios with longitudinal and time course data, and for each scenario we define a suitable test of conditional independence.

- **The target variable is time-varying**. In this scenario the task consists of identifying the predictors that are associated with the outcome of interest *in the course of time*. An example is modeling how a gene expression progresses over time on the basis of other gene expressions. Missing values can occur, or not all subjects may have measurements for all time points (unbalanced design). This case can be further subdivided in two sub-scenarios: the **Temporal-longitudinal** scenario, the same samples are being studied at all time points (longitudinal data), and the **Temporal-distinct** scenario, where different samples are being studied for each time points. The latter typically arises when it is impossible to repeat the measurements on the same sample: prototypical examples are animal studies where specimens are killed for collecting internal organs at different time points.
- **The target variable is a static (non-temporal) variable**. In some studies the predictors are measured over time, however the dependent variable is static. An example is the study of gene-expressions differences between two mice groups (target). The task in this case is to identify the minimal set of genes whose trajectories, considered together, allow to best discriminate between the two groups. Also for this scenario we can identify two sub-cases, namely the **Static-longitudinal** scenario, where the same samples are measure over time, and the **Static-distinct** scenario, where different samples are considered at each time point.

**Fig. 1:**
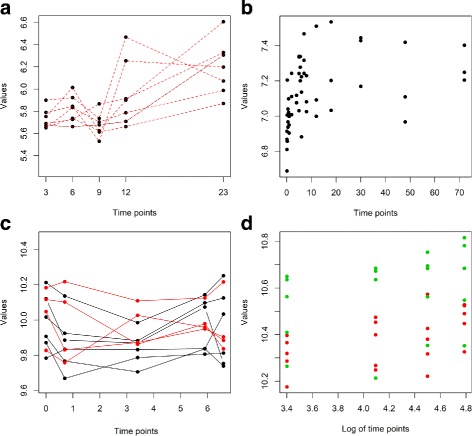
Graphical representation of the four different scenarios. In all panel the x-axis reports the time dimension, while y-axis reports the log-transformed expression value of a randomly-chosen probeset from one of the datasets used in the experimentation. **a** Temporal-longitudinal scenario. All data, including the target variable, consists of longitudinal (repeated) measurements. Values from the same subject are linked with a dashed line. (data from the GDS3915 dataset). **b** Temporal distinct scenario. Each observed value refers to a different subject (data from the GDS964 dataset). **c** Static longitudinal scenario. There are two groups (red and black lines), and each group consists of trajectories of longitudinal measurements. Each trajectory refers to the same subject (data from the GDS4146 dataset). **d** Static distinct scenario. At every time point different subjects are measured. Green and red colors indicate the two populations from which the subjects are sampled from (data from the GDS2456 dataset)

Figure 1 graphically presents these four scenarios using data from some of the real datasets used in our experimentation. More information and example data for each scenario are presented in the Additional file 1.

These scenarios represent the most common designs for biological studies involving temporal data, and are widely applied in other fields as well. Other scenarios/study designs are of course possible (for example measurements might be repeatedly taken for each sample at each time point), however we consider them less relevant and out of the scope of the present paper.

In this paper we use the Statisticaly Equvialent Signatures (SES) algorithm [5, 10] as a prototype for the class of constraint-based algorithms. The predictors selected by SES (signature) are the neighbors of *T* in any BN (faithfully) representing the data distribution. This is a subset of the full Markov Blanket but it has been shown to be a good approximation for predictive purposes in extensive empirical studies [11]. Some algorithms (HITON, MMMB) do continue in trying to identify the full Markov Blanket which also includes the spouses of *T* at the expense of computational time. SES can successfully scale up to cases where *p*>>*n*, preserving excellent predictive capabilities [5]. We measure the time complexity of the algorithm in terms of the number of performed conditional independence tests. Each variable must be contrasted against each subset of the selected signature before being eliminated. This would require a number of tests in the order of *O*(*p*·2^*s*^), where *p* is the number of variables and *s* the number of selected variables. However, we only allow conditioning upon maximum *k* variables at the time, decreasing the complexity of the algorithm to *O*(*p*·*s*^*k*^). This means that the algorithm can still require an exponential number of tests with respect to the size of the selected signatures; however, in our experience the actual computational requirements of the algorithm are much lower, also due to the parsimonious signature often retrieved.

A desired feature of SES is the fact that it heuristically and efficiently attempts to identify statistically, equivalent solutions, i.e., minimal-sized feature subsets with the same optimal predictive performance. As mentioned before, when the distribution is faithful to a BN the solution is unique; however, in practice whether due to finite sample or deviations from assumptions there are multiple (empirically) equivalent solutions. Identifying all equivalent solutions is important when feature selection is employed for knowledge discovery and getting insight to the domain under study. Returning an arbitrarily-chosen single solution *S* may mislead the domain expert into thinking that all other variables are either redundant or irrelevant, when the situation can be reversed if selecting some other feature subset *S*^′^.

In our empirical study, we compare SES against the state-of-the-art feature selection algorithms for the above 4 scenarios on gene-expression data. SES successfully scales up to tens of thousands of gene trajectories. In terms of selection quality and predictive performance, SES outperforms other methods in the Temporal-longitudinal scenario, is on par or better in the Static-longitudinal and Static-distinct scenarios while selecting many fewer variables, while it is outperformed in the Temporal-distinct scenario.

The rest of the paper is organized as follows. The “Methods” section introduces conditional independence testing for temporal data, as well as the SES algorithm. A comparative evaluation of the proposed approaches against LASSO-inspired algorithms is then performed on real, high dimensional omics data. Discussion and conclusions end the paper.

### Related work

In general, variable selection algorithms can be classified into two main categories, filter based and wrappers [12]. Methods of the first class select a subset of relevant features independently of the modeling algorithm that will be subsequently applied. On the other hand, wrapper methods try to select the set of features that optimize the performance of a specific classifier. A large bulk of literature has been published on the subject, with methods using several different approaches [13–23].

Finally, embedded methods are modeling algorithms whose operation automatically lead to the selection of the most relevant features (e.g., classification and regression trees [24]).

Many variable selection methods for classification of high dimensional biological data (particularly gene expression) have been proposed in the last decades [25]. For a recent review and open problems with regard to variable selection in high dimensional data the reader is addressed to Bolón-Canedo et al. [26].

In this work, we have carefully reviewed the current literature for identifying the most related and recent variable selection methods suitable for the four scenarios depicted above. Particularly, we have sought methods both applicable on temporal data and scalable to high-dimensional problems (i.e, thousands of candidate predictors).

In brief, the *glmmLasso* algorithm seems to be the most well-performing method for studies that belong to the Temporal-longitudinal scenario, according to the comparison performed in [27]. This algorithm combines mixed-models representation of complex variance structures with the sparsity of LASSO solutions; as a drawback, the resulting model is non-convex and difficult to optimize. In the Temporal-distinct and static-distinct scenarios there is no within-sample variance, and these two cases can be addressed with variable selection algorithms designed for non-temporal data. The Static-longitudinal scenario corresponds to discriminant analysis in longitudinal data, and not much research has been performed in the context of variable selection, see for example [28–30].

### Available approaches for the Temporal-longitudinal scenario

Several approaches for variables selection were proposed in the last 15 years for studies where both the outcome and the predictors are measured over time on the same samples. Most of these approaches use either Generalized Linear Mixed Models (GLMM) or Generalized Estimating Equations (GEE).

On GLMM, Ni et al. [31] proposed a double-penalized likelihood approach in semi-parametric mixed models. Bondell and co-authors [32] proposed an algorithm that performs simultaneous selection of the fixed and random factors using a modified Cholesky decomposition and maximum penalized likelihood estimation, along with the smoothly clipped absolute deviation (SCAD). A similar approach, using adaptive LASSO penalty functions instead of SCAD, was presented as well [33]. Zhao et al. [34] suggested using a basis function approximations and a partial group SCAD penalty for semi-parametric varying coefficient partially linear mixed models, while Tang et al. [35] focused on quantile varying coefficient models via penalizing the *L*_*γ*_ norm. Schelldorfer et al. [36] proposed an *L*_1_-penalty term for linear mixed models, and this work was later extended to include Poisson and binary logistic regression [37]. A method quite similar to the one of [37] was proposed in [27]; however the latter uses a gradient ascent algorithm whereas the former uses a coordinate gradient descent method based on a quadratic approximation of the penalized log-likelihood. Finally, a comparison of model selection methods for linear mixed models based on four major approaches is presented in [38]: information criteria such as AIC or BIC, shrinkage methods based on penalized loss functions such as LASSO, fence (ad-hoc procedures) and Bayesian techniques.

The literature is less extensive when it comes to GEE. The use of a modified AIC, termed quasi-likelihood information criterion (QIC), was proposed in [39]. Cantoni and co-authors [40] first used a generalised Mallow’s criterion, and subsequently [41] used a Markov chain Monte Carlo (MCMC) procedure for variable selection without visiting all possible candidate models. The case of missing-at-random data was addressed in [42] by using a missing longitudinal information criterion selecting the optimal model and the correlation structure. Finally, a penalized GEE method that is consistent even when the working correlation structure is misspecified was presented in [43].

Some Bayesian techniques include [44–46] among others. The first used a Cholesky decomposition of the random effects covariance matrix and introduced a further decomposition of the Cholesky decomposed lower triangular matrix. The elements of the resulting diagonal matrix are assigned zero-inflated truncated-Gaussian priors and MCMC methods are applied. However, these types of approaches are discouraged [47], as they are computationally heavy and are prior dependent. Han and co-authors [45] compared a number of methods for comparing two linear mixed models using Bayes factors. They also mentioned that these kinds of methods require substantial human intervention and high computational power.

A common drawback of all the procedures presented so far is that they are applicable only on a small number of candidate predictors. The only exceptions are presented in [35–37], [43], that were tested on 100, 200, 500 and 1000 candidate predictors in their respective simulation studies. To note, these studies do not report information about the computational time required by the algorithms. Moreover, authors do not usually provide implementations of the methods they propose. The only methodologies available as R packages are the one presented by [36], under the name GLMMLasso, and the *glmmLasso* package by [27], which offers linear, Poisson and binary logistic mixed models.

### Available approaches for the static-longitudinal scenario

This scenario refers to the task of discriminant analysis in longitudinal data. According to the concise review presented in [48], variable selection is somewhat not heavily researched in this context. More recently, L_1_ type constrains such as LASSO and SCAD allowing for grouped variables [28] were suggested. Matsui et al. [49] extended previous work to include multinomial logistic regression where the variables are selected in a grouped way. Finally, approaches based on functional regression also exist in the literature, see for example [50].

### Available approaches for the Temporal-distinct and Static-distinct scenarios

Both the Temporal-distinct and Static-distinct scenarios are defined over time-course data measured at each time point on different samples. Thus, the within-sample variance cannot be modeled for these scenarios. This allows variable selection methods devised for non-temporal data, as the widely used LASSO [51], to be applied in this context.

The LASSO algorithm started gaining popularity after the work in [52] who suggested the least angle regression as a better and faster way to solve its underlying optimization problem. A coordinate descent algorithm, which allows using the LASSO penalty in the context of generalized linear models was then suggested [53]. This latter approach is implemented in the R package *glmnet* [54].

Grouped Lasso (gLASSO, [55]) was developed to handle categorical predictors, which are often encoded in linear modeling as groups of binary variables (dummy variables). For the sake of consistency, the dummy variables corresponding to a single categorical predictor should be either included or excluded altogether (“as a group”) in the final LASSO solution. More recently, a quite efficient gLASSO implementation was proposed by [56], with their code made available in the R package *gglasso* [57].

## Methods

In this section we discuss in detail how to adapt constraint-based method for temporal data analysis. First, we will briefly present Generalized Linear Mixed Models (GLMM) and Generalized Estimating Equations (GEE). Both techniques are suitable for devising conditional independence tests for temporal data with (un)balanced study designs. For a thorough comparison between GLMM and GEE see [58, 59].

### Generalised linear mixed models

Let **T**_*i*_ denote the *n*_*i*_-dimensional vector of observed values for the target (response) variable *T* in the *i*-th subject at the different *d* time-points. We model the link of **T**_*i*_ with *p* covariates via the following equation:

1 $$ g\left(\mathbf{T}_{i}\right) = \mathbf{X}_{i}\pmb{\beta} + \mathbf{W}_{i}\mathbf{b}_{i} + \mathbf{e}_{i}, \ \ \ i= 1, \ldots, K.  $$

The vector *β* is the (*p*+1)-dimensional vector of coefficients for the *n*_*i*_×(*p*+1) fixed effects design matrix **X**_*i*_, which contains the predictor variables. The vector **b**_*i*_∼*N*_*q*_(**0**,*Σ*) is the *q*-dimensional vector of coefficients for the *n*_*i*_×*q* random effects matrix **W**_*i*_, while *Σ* is the random-effects covariance matrix. The vector $\mathbf {e}_{i} \sim N_{n_{i}}\left (\mathbf {0}, \sigma ^{2}\mathbf {I}_{n_{i}}\right)$ is the *n*_*i*_ dimensional within-group error vector which follows a spherical normal distribution with zero mean vector and fixed variance *σ*^2^.

We used the exchangeable or compound symmetry (CS) structure on the covariance matrix *Σ*. We decided not to use a first order autoregressive covariance (AR(1)) structure as a hyper-parameter of the GLMM method, since this type of structure did not improve the performance of generalised estimating equations (presented below) and would add a high computational burden to the fitting of GLMM.

*K* stands for the number of subjects and the total sample size (number of measurements) is equal to $N=\sum _{i=1}^{K}n_{i}$. The link function *g* connects the linear predictors on the right hand side of (1) with the distribution of the target variable. Common link functions are the identity, for normally distributed target variables, and the logit function for binomial responses.

The possibility of specifying random effects allows mixed models to adequately represent between and within-subject variability, and to model the deviates of each subject from the average behavior of the whole population. These characteristics make GLMMs particularly suitable for temporal and longitudinal data [9].

### Generalised estimating equations

Generalised Estimating Equations (GEE), developed by [60, 61], are an alternative to mixed models for modeling data with complex correlation structures. In contrast to GLMM which are subject specific, GEE contain only fixed effects and thus are population specific.

Using the notation defined in the previous section, in GEE the *p* covariates are related to the outcome as

2 $$ g\left(\mathbf{T}_{i}\right) = \mathbf{X}_{i} \pmb{\beta} + \mathbf{e}_{i}, \ \ \ i = 1, \ldots, K.  $$

with the variance of the response variable *T* being modeled as *Var*(*T*_*ij*_)=*ϕ*·*α*_*ij*_, *j*=1…*n*_*i*_, where *ϕ* is a common scale parameter and *α*_*ij*_=*α*(*T*_*ij*_) is a known variance function. We will focus on two different correlation structures for estimating *α*, the CS and the first order autoregressive AR(1):

3 $$  \begin{aligned} \text{CS:} \quad\text{Cor}\left(\mathbf{T}_{ij}, \mathbf{T}_{ij'}\right) & =\,\, \alpha \\ \text{AR(1):} \quad\text{Cor}\left(\mathbf{T}_{ij}, \mathbf{T}_{ij'}\right) & =\,\, a^{\left|j-j' \right|}. \end{aligned}  $$

CS assumes that correlations of measurements for the same subject at different time-points are always the same, regardless of the temporal distance between them. Depending on the specific application, this might be not very realistic. In contrast, the AR(1) structure assumes that the correlation between measurements at different time points for the same subject decreases exponentially as the temporal gap between them increases.

A precise numerical estimation of *α* is critical in GEE modeling; we use the jackknife variance estimator suggested by [62], which is quite suitable for cases when the number of subjects is small (*K*≤30), as in many biological studies. The simulation studies conducted by [63] and [64] showed that the approximate jackknife estimates are in many cases in good agreement with the fully iterated ones.

### Conditional independence tests for the Temporal-longitudinal scenario

We devise two independence tests based on GLMMs (Eq. 1) and GEEs (Eq. 2) respectively. This scenario assumes the predictors and the target variable are measured at a fixed set of time-points *τ*={*τ*_1_,…,*τ*_*m*_} in the same set of subjects. For balanced designs, all subjects are measured at all time-points, i.e, *n*_*i*_=*n*,∀*i*. The target variable is often a gene-expression trajectory and thus, in the rest of the paper and for this scenario we assume a continuous target.

Recall that the null hypothesis *Ind*(*X*;*T*|**Z**) implies that *X* is not necessary for predicting *T* when **Z** is given, and thus the conditional independence tests can be thought of as a testing the significance of the coefficient of *x*. The null and full models are written as

4 $$  \begin{aligned} H_{0}: \mathbf{T}_{i} =\,\, \mathbf{1} a \,\,+ \mathbf{1} b_{i} \,\,+ \,\, \gamma \pmb{\tau} \,\,+ \,\, \pmb{\delta} \mathbf{Z}_{i} & \\ H_{1}: \mathbf{T}_{i} =\,\, \mathbf{1} a \,\,+ \mathbf{1} b_{i} \,\,+ \,\, \gamma \pmb{\tau} \,\, + \,\, \pmb{\delta} \mathbf{Z}_{i} &\quad + & \beta \mathbf{X}_{i} \end{aligned}  $$

where **1** is a vector of 1s, *a* is the global intercept, *b*_*i*_ stands for the random intercept of the *i*-th subject, *γ*, *δ* and *β* are the coefficient of the predictors, and the generic link function *g*(.) (Eq. 1) has been substituted with the identity one.

This formulation stems from two specific modeling choices: (a) we use the vector of actual time points *τ* as a covariate, in order to model the baseline effect of the time on the trajectory of the target variable. Time becomes a linear predictor of the target. Other choices are possible, but would require more time-points that are typically not available in gene-expression data. (b) We include random intercepts, meaning we allow a different starting point for the estimated trajectory of each subject. This choice leads to **W**_*i*_=**1**_*n*__*i*_,∀*i*, where **1**_*n*_ is a vector of ones of size *n*. However, we do not allow random slopes, thus assuming all subjects have the same dynamics. This choice was dictated by the need of avoiding model over-specification, especially considering the small sample size of the datasets used in the experimentation.

Pinheiro and Bates [9] suggests the use of the *F*-test for comparing the two models, where only the model, the full, under the alternative is fitted and the significance of the coefficient *β* is tested. Another possible choice would be the log-likelihood ratio test, however the *F*-test is preferable for small samples, since the type I error is better controlled with the *F* distribution.

A second test is based on the GEE model. The null and alternative models now lose the random terms:

5 $$ \begin{aligned} H_{0}: \mathbf{T}_{i} = \,\, \mathbf{1} a &\quad + & \gamma \pmb{\tau} &\quad + & \mathbf{Z}_{i} \pmb{\delta} & & \\ H_{1}: \mathbf{T}_{i} = \,\, \mathbf{1} a &\quad + & \gamma \pmb{\tau} &\quad + & \mathbf{Z}_{i} \pmb{\delta} &\quad + & \beta \mathbf{X}_{i} \end{aligned}  $$

GEE fitting does not compute a likelihood [59] and thus, no log-likelihood ratio test can be computed. A Wald test is used instead here again and the significance of the coefficient *β* is tested. Because of the lack of likelihood computation, its effectiveness in assessing conditional independence is questionable [65]. Despite these theoretical considerations, the experimental results proved the test to be quite effective in our context.

### Conditional independence tests for the Static-longitudinal scenario

The Static-longitudinal scenario assumes longitudinal data with continuous predictors and a static target variable *T* that is either binary or multi-category. The goal is to discriminate between two or more groups on the basis of time-depending covariates. As in the Temporal-longitudinal scenario, the presence of longitudinal data requires to take into account the within-subject correlations.

We have devised a two-stage approach, partially inspired by the work of [66] and [67], for testing conditional independence in this scenario. In our approach a separate regression model is first fitted for each subject and predictor, using the time-points vector *τ* as unique covariate:

6 $$  \mathbf{G}_{i} = \gamma_{i0} + \gamma_{i1}\pmb{\tau}, \ \ \ i = 1, \ldots, n.  $$

Here, **G**_*i*_ is the vector of measurements for subject *i* and the generic predictor variable *G*. At the end of this step we end up with a matrix ***Γ*** with dimensions *K*×(2·*p*), containing all coefficients derived with the *K* models specified in (6). The two nested models needed for testing conditional independence can then be specified as:

7 $$  \begin{aligned} H_{0}: g\left(\mathbf{T}_{i}\right) = \,\, \mathbf{1} a &\quad + & \pmb{\delta} \pmb{\Gamma}_{\mathbf{Z}} \\ H_{0}: g\left(\mathbf{T}_{i}\right) = \,\, \mathbf{1} a &\quad + & \pmb{\delta} \pmb{\Gamma}_{\mathbf{Z}} &\quad + & \pmb{\beta} \pmb{\Gamma}_{X} \\ \end{aligned}  $$

where *Γ*_**Z**_ are the coefficients corresponding to the set of conditioning variables **Z** and *Γ*_*X*_ are the coefficients corresponding to the variable *X*. A logit function *g*(.) is used for linking the linear predictors to the binomial (or multinomial) outcome. The log-likelihood ratio test (calibrated with a *χ*^2^ distribution) is used to decide which of the two models is to be preferred.

### Conditional independence tests for the Temporal-distinct and Static-distinct scenarios

In these two scenarios different subjects are sampled at each time point (time-course data), and subject-specific correlation structures cannot be modeled. For the Temporal-distinct scenario, where the target variable is continuous, it is thus possible to use models (5) for assessing conditional independence. In absence of subject-specific correlation structures the GEE models reduce to standard linear models that can be compared with the standard *F*-test. A similar approach can be used for the Static-distinct scenario, where the outcome is binary or multinomial, by using a logit link function instead of the identity.

### The SES algorithm

First introduced in [10], the SES algorithm attempts to identify the set(s) of predictors (signatures) that are minimal in size and provide optimal predictive performances for a target variable *T*. The basic idea is that if ∃**Z**, s.t., *Ind*(*X*;*T*|**Z**), then *X* is superfluous for predicting *T*. Thus, SES repetitively applies a test of conditional independence until it identifies the predictors that are associated with *T* regardless of the conditioning set used. Under certain conditions, these variables are the neighbors of *T* in a Bayesian Network representing the data at hand [2]. An interesting characteristic of SES is that it can return multiple, statistically indistinguishable predictive signatures. As discussed in [68], limited sample size, high collinearity or intrinsic characteristics of the data may produce several signatures with the same size and predictive power. From a biological perspective, multiple equivalent signatures may arise from redundant mechanisms, for example genes performing identical tasks within the cell machinery. The SES algorithm is further explained in the Additional file 1 and in [5].

#### Equipping constraint-based methods with conditional independence test for temporal data

SES belongs to the class of constraint-based feature-selection methods [4]. This type of algorithm processes the data exclusively through tests of conditional independence that assess *Ind*(*X*;*T*|**Z**). *This means that in order to extend any constraint-based methods to temporal data it is sufficient to equip an appropriate test, such as the ones defined in Eqs.*(4)-(7).

### Experimentation on real data

The experimental evaluation aims at assessing the capabilities of the proposed conditional independence tests in real setting. For each scenario we identified several gene-expression datasets over which we applied the SES algorithm equipped with the conditional independence test most suitable for the data at hand. The feature subsets identified by SES were then fed to modeling methods for obtaining testable predictions.

Furthermore, in each scenario we contrasted SES against a feature selection algorithm belonging to the family of LASSO methods. This class of algorithms has proven to be well-performing in several applications, including variable selection in temporal data (see the Section regarding the literature review). Particularly, we compare against glmmLasso [27] for the Temporal-longitudinal scenario, with standard LASSO regression [51] for the Temporal-distinct scenario, and the grouped LASSO (GLASSO) for classification [54, 56] in the Static-longitudinal and Static-distinct scenarios.

We excluded from this comparative analysis approaches that a) do not scale-up to thousands of variables (e.g., Bayesian procedures), b) require a number of time points much larger than the applications taken into consideration in this work (as for functional regression, [69]), and c) in general do not have available implementations.

The configuration settings of all algorithms involved in the experimentation were optimized by following an experimentation protocol specifically devised for estimating and removing any bias in performance estimation due to over-fitting.

### Datasets

We thoroughly searched the Gene Expression Omnibus database (GEO, http://www.ncbi.nlm.nih.gov/) for datasets with temporal measurements. Keywords “longitudinal”, “time course”, “time series” and “temporal” returned nearly 1000 datasets. We only kept datasets having at least 15 measurement and at least three time points, and complete information about the design of the study generating the data. This resulted in at least 6 datasets for each scenario, except for the Static-longitudinal scenario, where we identified 4 datasets with at least 8 measurements. Detailed information on the selected datasets are available in the (Additional file 1: Tables S5 and S6).

### Modeling approaches

For the Temporal-longitudinal scenario SES was coupled with either GLMM or GEE regression, so as to mirror the conditional independence test equipped to the algorithm. The glmmLasso algorithm is used for comparison, using a model similar to (4) defined over the whole predictors matrix **X**

8 $$ \begin{aligned} \mathbf{T}_{i} = &\quad \mathbf{1}a + b_{i} & + \quad& \gamma \pmb{\tau} & + \quad& \mathbf{X}_{i} \pmb{\beta}\\ \end{aligned}  $$

For the Static-longitudinal scenario, logistic or multinomial regression was applied on the columns of the matrix *Γ* selected by SES, depending on the outcome at hand. The grouped Lasso (GLASSO, [56]) algorithm was used for comparison. GLASSO allows to specify groups of variables that can enter the final model only altogether. Particularly, the GLASSO was applied on the whole matrix *Γ*, forcing the algorithm to either select or discard predictors in pairs, following the way columns in *Γ* correspond to the original predictors.

For Temporal-distinct and Static-distinct scenarios SES was always coupled with standard linear, logistic or multinomial regression (depending on the specific outcome), while the standard LASSO algorithm (binary outcome) and GLASSO (multinomial outcome) were used for comparison (see Additional file 1 for further details).

In all analyses SES’ hyper-parameters maximum conditioning variables size *k* and significance level *a* varied between {3,4,5} and {0.05,0.1}, respectively. The *λ* penalty values generated by the Least Angle Square (LARS) algorithm [52] were used for the LASSO models of all scenarios, apart from the temporal-longitudinal. LARS cannot be adapted to this latter scenario, and thus the range of values was separately determined for each dataset, by using all integer values between *λ*_*min*_, the smallest value guarantying the invertibility of the Hessian matrix in each fold, and *λ*_*max*_, the highest value after which no variable was selected.

### Experimentation protocol

We used the *m*-fold cross-validation procedure with the Tibshirani-Tibshirani (TT) bias correction [70] for model selection and performance evaluation. In the standard cross-validation protocol the available samples are partitioned in *m* folds, with approximately an equal number of samples each. Each fold is in turn held-out for testing, while the remaining data form the training set. The current modeling approach is applied several times on the training set, once for each predetermined configuration setting, and the predictive performances of the corresponding models are evaluated on the hold-out fold. The configuration with the best average performance is then used for training a final model on the whole dataset. In all experimentation *m* was set to either 4 or 5, so that to have at least two measurements in each fold. Particularly, folds correspond to one or more subjects in the Static-longitudinal scenario, and to one or more time points in the other scenarios.

The performance of the best configuration is known to be optimistically biased, and thus a correction is needed for a fair evaluation. The TT method is a general methodology for estimating and removing the optimistic cross-validation bias. If the performance’s metric is defined in terms of prediction error (the lower the error the better the performance), the bias estimation according to the TT method is the following:

9 $$  \hat{\text{bias}}=\frac{1}{m}\sum_{i=1}^{m}\left[e_{i}\left(\hat{\pmb{\theta}} \right) - e_{i}\left(\hat{\pmb{\theta}}_{i} \right) \right],  $$

where *e*_*i*_ is the performance on fold *i*, while $\hat {\pmb {\theta }}$ and $\hat {\pmb {\theta }}_{i}$ are the configurations corresponding to the best average performance and to the best performance of the *i*-th fold, respectively. Signs in (9) should be interchanged if the performance metric assigns higher scores to better models.

The statistical significance of the difference between average performances is computed through permutation-based t-tests, where single performances are randomly permuted for approximating the null distribution.

All of the simulations, computations and time measurements were performed on a desktop with Intel Core i5-3470 CPU @ 3.2 processor, 4 GB RAM memory using a 64-bit R version 3.2.2.

## Results and discussion

### Coupling SES with GLMM and GEE

First, we contrasted the performances of GLMM and GEE-based conditional independence tests in the context of the Temporal-longitudinal scenario. Table 1 reports the results of the comparison.

**Table 1:** Temporal-longitudinal scenario: comparison between SES equipped with GLMM (SESglmm) and SES equipped with GEE

Dataset	MSPE			Average time (in seconds)		
	SESglmm	SESgee(CS)	SESgee(AR(1))	SESglmm	SESgee(CS)	SESgee(AR(1))
GDS5088	0.131 (0.000)	0.189 (0.1)	0.289 (0.018)	1562.51 (230.53)	1022.45 (217.99)	933.14 (180.34)
GDS4395	0.116 (0.007)	0.156 (0.019)	0.298 (0.028)	21167.21 (26089.48)	4862.15 (1724.89)	5577.80 (1890.15)
GDS4822	0.066 (0.000)	0.055 (0.001)	0.045 (0.004)	1785.66 (321.92)	2103.96 (490.74)	1492.30 (205.03)
GDS3326	0.062 (0.001)	0.052 (0.000)	0.063 (0.002)	6617.09 (472.16)	3167.78 (795.74)	2348.69 (390.10)
GDS3181	0.805 (0.096)	0.458 (0.000)	0.458 (0.00)	1684.90 (206.26)	1011.44 (152.59)	748.18 (105.32)
GDS4258	0.074 (0.000)	0.149 (0.003)	0.152 (0.002)	4135.76 (506.15)	2818.024 (418.97)	2078.52 (462.30)
GDS3915	0.527 (0.038)	0.553 (0.01)	0.439 (0.000)	669.18 (63.93)	511.82 (84.22)	491.91 (108.64)
GDS3432	0.057 (0.001)	0.060 (0.008)	0.038 (0.003)	3275.22 (474.06)	2213.11 (371.68)	2104.05 (546.76)
Average	0.230 (0.280)	0.209 (0.192)	0.223 (0.172)	5112.2 (6756.04)	2378.56 (1566.36)	1971.82 (1611.13)

The latter is indicated as SESgee(CS) and SESgee(AR(1)), depending by the employed variance estimator. TT-corrected, cross-validated mean square prediction error are reported for each dataset, along with their standard deviation (in parenthesis). Average (standard deviation) computational time is reported as well, while the last line reports performances averaged across datasets. The MSPE values are not statistically different, however SESgee(AR(1)) is faster than the other alternatives

For each dataset the cross-validated, TT-corrected Mean Squared Prediction Error (MSPE) is reported (standard deviation in parenthesis), along with the respective computational time in Table 1. Average performances are reported at the bottom line. Methods are indicated as SESglmm, SESgee(CS)) and SESgee(AR(1)), corresponding to SES coupled with GLMM and GEE, the latter using either the CS or AR(1) covariance structure. All methods obtain statistically equivalent results in terms of MSPE (all paired permutation-based t-test *p*-values are above 0.37). The average computational time largely varies, with SESgee(AR(1)) being the fastest of the three methods (all paired permutation-based t-test *p*-values are below 0.002). For all methods, computational times strongly depend upon the number of variables of each dataset, in a log-linear way (see Additional file 1).

Since the three versions produced equally predictive results, in the remaining of the analysis we use only SESglmm, in order to ensure a comparison as fair as possible with the GLMM based method glmmLasso.

### glmmLasso scalability in high-dimensional data

**Fig. 2:**
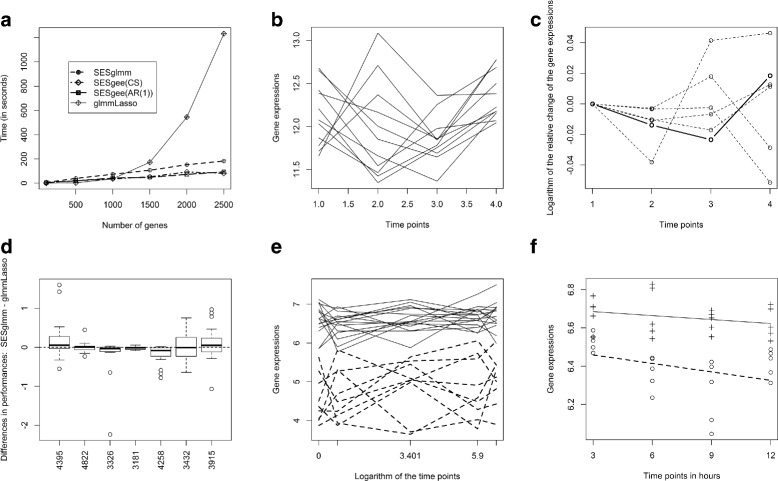
**a** Temporal-longitudinal scenario: Time in seconds required by glmmLasso and SES equipped with different conditional independence tests on the GSD5088 dataset. The number of randomly selected predictors is reported on the x-axis, while y-axis reports the required computational time: glmmLasso rapidly becomes computationally more expensive than any SES variant. **b** Gene expression over time for the target gene CSHL1 in dataset GDS5088 (one line for each subject). **c** Average relative change for the target gene and predictors reported in model 10. The expression of the genes was averaged over subjects for each time point, and the logarithm of the change with respect to the first time point was then computed. The target gene appears as bold line, whereas the 5 predictor genes are reported as dashed lines. **d** Differences in performance between SESglmm and glmmLasso for the 20 replications on each dataset. Negative values indicate SESglmm outperforming glmmLasso; SESglmm is always comparable or better than glmmLasso, especially in dataset GDS5088 (excluded for sake of clarity). **e** Static-longitudinal scenario: Expressions over time of gene TSIX, selected by SES for dataset GDS4146. The plot show one line for each subject: there is a clear separation between the two classes included in the dataset (dashed and solid lines, respectively). **f** Static-distinct scenario: Expressions over time of gene Ppp1r42, selected by SES for dataset GDS2882. The dotted and dashed lines correspond to the average trend of the gene in two different classes; differences in intercept and trend are easily noticeable

Preliminary analyses pointed out glmmLasso’s limited ability of efficiently (in computational terms) scaling up to a few thousands of predictors (glmmLasso’s implementation is limited to 17,000 variables). We characterized glmmLasso scalability by running the algorithm on increasingly larger numbers of randomly selected variables. Figure 2a reports the results obtained from dataset GSD5088. Different lines report time performances of glmmLasso, and SES equipped with different conditional independence tests. glmmLasso requirements in terms of computational time increase in a super-linear way with the number of predictors (see Additional file 1: Figures S1 and S2 for time comparisons with all datasets). An interesting feature of the SES implementation that is worthy to mention is the fact that information about the univariate associations (test statistics and associated *p*-values) is stored. Hence, when the hyper-parameters change values, the algorithm begins from the second step. For the 6 pairs of configurations (pairs of *a* and _*k*_) used in our experimental analysis this results in a significant amount of computational savings.

The same analysis was repeated on all datasets selected for the Temporal-longitudinal scenario, consistently achieving similar results (Additional file 1). Consequently, for each dataset related to the Temporal-longitudinal scenario only 2000 randomly selected predictors were retained in all subsequent analyses, so that the experimentation could be performed in a reasonable time and to allow a fair comparison between SESglmm and glmmLasso (see Additional file 1: Table S9 for the values of the penalty parameter used in glmmLasso).

### Results on the four scenarios

Table 2 reports the main results of the experimentation. For each dataset, cross-validated, TT-corrected performances are reported as *average (st.d.)*. Zero standard deviations are caused by numerical rounding. For the Temporal-longitudinal and Temporal-distinct scenarios the MSPE metric is used, with lower values indicating better performances, while the Percentage of Corrected Classification (PCC) metric is used for the other scenarios, with higher values indicating better performances. Average differences (SES - LASSO) over all datasets are reported for each scenario and statistically significant differences at 0.01 and 0.05 significance level are indicated with ^∗∗^ and ^∗^, respectively.

**Table 2:** Cross-validated, TT-corrected performances of SES and LASSO-type methods on the four scenarios

Temporal-longitudinal scenario					Temporal-distinct scenario				
	MSPE		Selected vars			MSPE		Selected vars	
Dataset	SESglmm	glmmLasso	SESglmm	glmmLasso	Dataset	SES	LASSO	SES	LASSO
GDS5088	**0.081** (0.026)	0.160 (0.042)	5.25 (0.85)	5.15 (8.65)	GDS3859	0.068 (0.006)	**0.019** (0.002)	3.5 (0.51)	11.81 (4.66)
GDS4395	**0.104** (0.041)	0.640 (0.568)	5.37 (0.56)	12.35 (13.61)	GDS972	0.022 (0.000)	**0.001** (0.000)	5.83 (0.92)	22.2 (9.85)
GDS4822	**0.115** (0.484)	0.765 (0.436)	4.75 (0.85)	3.16 (5.77)	GDS947	0.056 (0.000)	**0.054** (0.026)	5.92 (0.65)	12.40 (5.40)
GDS3326	**0.135** (0.021)	0.234 (0.139)	5.42 (0.78)	2.42 (7.45)	GDS964	0.033 (0.000)	**0.003** (0.000)	5.73 (0.69)	25.69 (11.86)
GDS3181	0.971 (0.484)	**0.684** (0.257)	4.17 (0.87)	0.35 (2.15)	GDS2688	0.184 (0.006)	**0.005** (0.001)	5.79 (1.06)	20.64 (10.93)
GDS4258	**0.234** (0.096)	9.882 (4.518)	3.83 (0.51)	1.48 (4.06)	GDS2135	0.053 (0.002)	**0.014** (0.003)	3.80 (0.76)	10 (5.72)
GDS3432	**0.357** (0.017)	2.283 (1.572)	1.67 (3.51)	0.08 (0.55)	Av. diff.	0.053 ^*a*^		-12.03 ^*a*^	
GDS3915	**0.059** (0.002)	0.150 (0.055)	5.12 (0.80)	1.66 (4.62)					
Av. diff.	-1.59 ^*b*^		1.12 ^*b*^						
Static-distinct scenario					Static-longitudinal scenario				
	PCC		Selected vars			PCC		Selected vars	
Dataset	SES	LASSO	SES	LASSO	Dataset	SES	GLASSO	SES	GLASSO
GDS4319	0.873 (0.000)	**0.995** (0.000)	2.1 (0.31)	8 (0.00)	GDS4146	**1.000** (0.000)	0.858 (0.142)	1.00 (0.00)	0.42 (1.38)
GDS3924	**0.729** (0.000)	0.528 (0.104)	2.75 (0.44)	53.56 (28.55)	GDS4518	**0.750** (0.000)	0.417 (0.333)	1.75 (0.44)	3.04 (2.15)
GDS3184	0.556 (0.067)	**0.578** (0.111)	3.00 (0.00)	10.62 (5.16)	GDS4820	0.500 (0.000)	**0.667** (0.167)	2.00 (0.00)	5.14 (3.19)
GDS3145	**0.953** (0.000)	0.594 (0.125)	1.5 (0.88)	0.6 (0.55)	GDS1840	**0.625** (0.000)	0.500 (0.250)	1.5 (0.51)	2.67 (2.03)
GDS2882	**0.800** (0.000)	0.750 (0.000)	1.5 (0.88)	0.25 (0.50)	Av. diff.	0.108		-1.23	
GDS2851	**0.722** (0.000)	0.694 (0.000)	2.25 (0.44)	0.75 (0.50)					
GDS1784	**0.861** (0.000)	0.694 (0.000)	1.75 (0.85)	0.5 (0.58)					
GDS2456	**1.000** (0.000)	0.739 (0.000)	1.2 (0.41)	0.44 (0.53)					
Av. diff.	0.115 ^*b*^		-6.52 ^*b*^						

For each dataset, performances are reported as *average (st.d.)*. Zero standard deviations are caused by numerical rounding. For Temporal-longitudinal and Temporal-distinct scenario’s performance are computed as Mean Squared Prediction Error (MSPE, lower values indicate better performances) and number of selected variables, while for the other scenarios the Percentage of Corrected Classification (PCC, the higher the better) is used instead of MSPE. The bold numbers indicate better performance; average differences over all datasets are reported for each scenario. Symbols ^*a*^ and ^*b*^ denote average differences that are statistically significant at 0.01 and 0.05, respectively. In terms of predictive performances, SES is always on par or better than LASSO type algorithms in all scenarios except for the Temporal-distinct

*On average, SES equipped with conditional independence tests for temporal data outperforms the corresponding LASSO algorithms, in terms of predictive performance, in all scenarios, except for the Temporal-distinct* scenario. We also note that LASSO methods did not select any variable in at least one fold of cross validation for several datasets, as indicated by an average number of selected variables < 1 (baseline predictive models are produced in these cases). When LASSO methods select at least one variable in each fold, their variability in number of selected variables is considerably higher than the one of SES. Particularly, for the Temporal-longitudinal scenario SESglmm largely outperforms, in terms of predictive performance, glmmLasso in all datasets except one (GDS3181), where glmmLasso is only marginally superior (See Additional file 1: Table S10). For the Temporal-distinct scenario the results are quite turned around, with LASSO having better predictive performances than SES, although at the cost of identifying larger and unstable sets of variables. Finally, SES generally outperforms LASSO in the Static-longitudinal and Static-distinct scenarios, both in terms of average PCC and number of selected features. No variables were selected for dataset GDS3944 by neither method, and thus we excluded this dataset from the results.

Since the results for the Temporal-longitudinal scenario could depend on the specific randomly selected gene used as target variable, we repeated the whole comparison for this scenario 20 more times, each time with a different target gene. Table 3 contains the respective results: for 4 out of 8 datasets SESglmm had statistically significantly better performance (on average), whereas for the other 4, the average performances did not differ in a statistically significant way. By aggregating the results we see that 91 out 160 times SESglmm had better performance than glmmLasso (i.e., 56.88*%* of the times, significantly larger than 50%, *p*-value=0.0395, according to the one-sided asymptotic z-test). Figure 2d shows the difference between SESglmm and and glmmLasso performances over the 20 repetitions as boxplots. GDS5088 is not shown for the sake of clarity: SESglmm largely outperforms glmmLasso for this dataset and the difference is so out-of-scale that would overshadow the differences in the other datasets (see Additional file 1: Figure S3).

**Table 3:** Temporal-longitudinal scenario: comparison between SESglmm and glmmLasso based on 20 replications with different target variable (gene) and independently randomly selected 2000 genes as predictor variables

Dataset	GDS5088	GDS4395	GDS4822	GDS3326	GDS3181	GDS4258	GDS3432	GDS3915
Average difference	-3.560(4.118)	0.188(0.516)	-0.003(0.134)	-0.180(0.506)	-0.020(0.04)	-0.139(0.288)	0.000(0.355)	0.093(0.455)
Proportion	19/20	7/20	9/20	13/20	15/20	10/20	10/20	8/20
*p*-value	0.0001 ^*a*^	0.128	0.938	0.0015 ^*a*^	0.0312 ^*b*^	0.024 ^*b*^	0.9946	0.3842

Average difference in performances (standard deviation of the differences appear inside the parentheses) and percentage of times SESglmm outperformed glmmLasso. The last line contains the permutation based *p*-value for the equality of the mean performances. Symbols ^*a*^ and ^*b*^ denote average differences that are statistically significant at 0.01 and 0.05, respectively. Notice that SESglmm is either statistically significantly better or on par with glmmLasso in terms of predictive performance

We give an example of how to interpret the models selected with SESglmm for Temporal-longitudinal datasets. Figure 2b reports the expression over time of the target gene CSHL1 for each subject in dataset GDS5088, while Fig. 2c shows the logarithm of the average relative change over time for the genes selected by SES as the best predictive signature for CSHL1. The fixed part of the corresponding mixed-effect model is

10 $$\begin{array}{@{}rcl@{}} {}\begin{array}{ll} CSHL1_{ij} = & 14.688 -0.092 \cdot \tau_{i} + 0.297 \cdot OR4G4P_{ij} \\ & -\, 0.628 \cdot RRNAD1_{ij} - 0.613 \cdot NDUFS2_{ij} \\ & +\, 0.212 \cdot {NC}_{ij} + 0.314 \cdot {ICMT}_{ij} \end{array} \end{array} $$

where *i*=1,2,3,4 represents the 4 time points, *j*=1,⋯,11 the 11 subjects, and *NC* stands for the genomic region *chr*1:232358622−232358886. The variance of the random intercepts is equal to 0.0018, corresponding to a 7.8*%* of the total variability. All coefficients are significant (maximum Wald test *p*-value 0.02), with positive coefficients indicating predictors whose trajectories over time agree with the one of the target genes, while the opposite holds for negative coefficients. The coefficient of the time effect has a negative sign; speculations on Fig. 2c suggest that a quadratic effect would perhaps perform better, but adding more parameters would easily lead to over-fitting, due to the limited number of time points and subjects. Figure 2e and f show the temporal trajectories of gene TSIX and Ppp1r42, respectively, which were included in the optimal predictive signatures of dataset GDS4146 (Static-longitudinal) and GDS2882 (Static-distinct). In both cases the trajectories of the two genes markedly differ between the two classes.

## Conclusions

In this work we described how constraint-based, feature selection methods can be extended for the analysis of (high dimensional) temporal data, by equipping them with suitable conditional independence tests. The main contribution of this work is thus indicating how a whole class of state-of-the-art, provably well-performing feature selection methods [4] can be easily extended to data characterized by (a) measurements taken over time and (b) high dimensionality, settings frequently encountered in biological studies as well as in other fields. Furthermore, conditional independence tests are the cornerstone of any constrained-based method for (causal) network reconstruction [71]; under this respect, this work also paves the path for extending this type of algorithms to temporal data.

We assessed the performances of the proposed approach by evaluating a prototypical constraint-based method, the SES algorithm, on several real-world gene expression datasets. Each dataset belongs to one out of four different scenarios, which represent common study designs for temporal data. The Temporal-longitudinal and Static-longitudinal scenarios represent longitudinal studies with time-dependent or static target variable, respectively, while the Static-longitudinal and Static-distinct scenarios refer to the case of different samples measured at each time point. The Temporal-longitudinal and Temporal-distinct scenarios required devising conditional independence tests able to take into account the idiosyncrasies of their respective data. The conditional independence test devised for the Temporal-longitudinal scenario addresses the within sample variation by employing the GLMM and GEE modeling techniques; the tests devised for the Static-longitudinal scenario uses a two-step regression strategy for addressing the problem of discriminant analysis in longitudinal data.

In the context of our experimentation, SES outperformed state-of-the-art methods belonging to the class of LASSO algorithms in three out of four scenarios. Particularly, in the Temporal-longitudinal scenario, SESglmm clearly superseded the glmmLasso algorithm [27]. Moreover, SESglmm easily scales to tens of thousands of variables, while glmmLasso computation requirements become rapidly prohibitive.

A key feature of the SES algorithm is its ability to produce multiple solutions, signatures, i.e. more than one set of predictor variables, which are statistically equivalent, as demonstrated in a recent publication [5]. Many times in biological studies, and not only, the outcome of the study, the final model, or the selected variables, is not what expected by the expert in the field. This could be justified by the fact that the chosen model is statistically equivalent to the expected model. And hence, a degree of miss-information has been delivered. Multicollinearity among the predictor variables should not be treated as a disease, but rather as a means of extracting extra information about the data.

We assessed the equivalence of the signatures produced by SES also in the context of this experimentation. For every signature computed by SES we fitted a predictive model and calculated the corresponding MSPE. The distribution of these MSPE values confirmed that SES produces signatures whose predictive value is close to each other (see Additional file 1: Tables S7, and S8 and Figures S7, S8 and S9 for details).

The results showed in “Results on the four scenarios” section and Table 2 purposely do not report information regarding the computational time. In the Temporal-longitudinal scenario SES’ computational requirements can be one or two orders of magnitude smaller than the ones of the glmmLasso. The opposite though is true for the other scenarios. A prototypical SES run on the GDS3859 dataset (45,100 variables and 23 samples, Temporal-distinct scenario) requires on average 278 s, while LASSO returns an answer in less than half a second. This difference is mainly due to implementation issues: the code of SES is written in R, while LASSO is based on a fast FORTRAN implementation. In addition, the SES algorithm needs to perform some additional computation for identifying multiple signatures. Other constraint-based methods, e.g., MMPC, that return only one signature are expected to be faster, in case the computational cost is an important parameter to consider.

Finally, we performed over-representation analysis on the pathways provided by the Kyoto Encyclopedia of Genes and Genomes (KEGG [72]) using the hyper geometric test. To ensure an adequate statistical power, we performed this analysis only for the 12 datasets where the probesets selected by SES correspond to five or more genes. For each dataset we used the pathways of the proper species. We found that on average 10 pathways are significant at FDR level 0.1 for each signature (the lists of enriched pathways are in the Additional file 1), meaning that the selected genes are significantly over-represented in known biological mechanisms. This indicates that performing feature selection with constraint-based methods coupled with conditional independence tests for temporal data can also provide biological insights, along with well-performing predictive models.

The main limitation of the present study is the relatively low sample size of several datasets, that makes difficult to precisely estimate performances. However, we note that the work presented by [73, 74] showed that the TT protocol is able to provide precise estimation even with relatively small sample size, computationally more efficiently than the more complex nested-cross validation protocol.

Future work will focus on several directions. Computationally, we have addressed the computational cost of the linear mixed models by using our implementations. We plan to add the other scenarios in this direction. Parallel computations for the first step of the algorithm will be made available for the other methods as well. More intriguing, the application of the tests introduced in this work on constraint-based, causal-discovery methods is currently under investigation.

## Additional file

Additional file 1 This file contains additional information regarding the datasets and the algorithms used in the paper as well as results on SES signatures equivalence and SES / Lasso computational requirements. (PDF 198 kb)
