# Supplementary material for: Feature selection for high-dimensional temporal data
Source: BMC Bioinformatics. 2018 Jan 23;19:17. doi: 10.1186/s12859-018-2023-7 (PMC5778658; doi:10.1186/s12859-018-2023-7)
Supplement: Additional file 1 — This file contains additional information regarding the datasets and the algorithms used in the paper as well as results on SES signatures equivalence and SES / Lasso computational requirements. (PDF 198 kb) [file 12859_2018_2023_MOESM1_ESM.pdf]

Feature selection for high-dimensional temporal data: supplementary material

Michail Tsagris   Vincenzo Lagani   Ioannis Tsamardinos

November 6, 2017

1 Example datasets for the 4 scenarios

- Table S1 give an example of the data structure in the **Temporal-longitudinal** scenario using the GDS4258 dataset. It can be seen that each subject contains 3 measurements in total, one for each of the 3 distinct time points.
- Table S2 presents example data from the GDS3859 dataset used in the **Temporal-distinct** scenario. Time points are again present, but each measurement refers to a different subject. This means that measurements can be freely shuffled within each column.
- An example of the **static-longitudinal** scenario is given in Table S3 where measurements are taken for each subject at three different time points. The goal is to discriminate between the two groups (infected - not infected). The numbers come from the GDS4518 dataset.
- Finally, data from the the GDS1784 dataset are used as an illustrative example of the **static-distinct** scenario in Table S4. Measurement across columns refer to different subjects, and thus also in this case measurements can be freely shuffled within each column.

Table S1: GDS4258 example data of the **Temporal-longitudinal** scenario.

	**Time points**		
**Subject**	4 Hours	18 Hours	48 Hours
1	6.28270	5.86563	6.57527
2	7.00407	7.40996	6.16624
3	6.66389	6.02764	6.30283
4	6.84890	7.04820	9.95038
5	9.28854	10.18260	9.64682

Table S2: GDS3859 example data of the **Temporal-distinct** scenario.

Time points			
0 Days	2 Days	4 Days	6 Days
6.458359	5.839970	6.123457	6.104052
5.509668	6.549998	6.376203	6.366128
6.071216	5.981081	6.287715	6.421593
6.586743	6.235236	6.064854	6.139346
6.166189	7.107606	6.852170	7.302294

Table S3: GDS4518 example data of the **Static-longitudinal** scenario.

Time points			
Subject and group	2 Hours	6 Hours	24 Hours
1-Not infected	10.4001	10.4665	10.3801
2-Not infected	10.9035	10.5774	10.5205
3-Not infected	10.3322	10.7477	11.0272
4-Infected	10.7066	11.0553	10.9070
5-Infected	10.9118	11.1279	10.7836
6-Infected	10.5297	10.4485	10.4771

Table S4: GDS1784 example data of the **Static-distinct** scenario.

	Time points				
Genotype	0 Hours	2 Hours	6 Hours	24 Hours	48 Hours
Wild type	7.038740	7.261660	6.771664	7.356893	7.168742
Wild type	7.092083	7.188458	6.699224	7.303311	7.197353
Wild type	7.043116	6.943344	77.299777	7.187256	7.088342
PKB alpha null	7.108351	6.902910	7.263736	7.288340	7.226173
PKB alpha null	7.106368	7.065784	7.303170	7.242576	7.038740
PKB alpha null	7.174931	6.998802	7.095330	7.254913	7.092083

2 The SES algorithm

The pseudo-code of SES is shown in Algorithm 1. Briefly, let $\mathcal{V}$ and T denote the set of predictor variables and the target variable respectively. In order to assess the null hypothesis $Ind(X;T|\mathbf{Z})$, a conditional independence test is performed. Denote with $p_{XT|\mathbf{Z}}$ the corresponding p-value. If $p_{XT|\mathbf{W}} \leq \alpha$, where α is a user-defined threshold, the null hypothesis is rejected. The algorithm requires to specify two hyper-parameters, k, the maximum size of the conditioning set, and α, the significance level for rejecting independence.

In the first step, the univariate (unconditional) associations between the target variable and the predictor variables are calculated. The variables corresponding to non significant associ-

ations are discarded. In all subsequent iterations conditional associations are calculated. At each iteration the algorithm identifies the variable with the highest association with T given any possible conditioning set $\mathbf{Z}$ s.t. $\mathbf{Z} \subseteq \mathcal{S}, |\mathbf{Z}| \leq k$, where k denotes the maximum number of conditioning variables. Variables found not associated with T for any conditioning set are discarded. SES stops when no variables are left for examination, i.e., $\mathcal{R} = \emptyset$. Before definitely discarding a variable, the algorithm checks whether the variable is equivalent to any predictor already in $\mathcal{S}$. If such equivalence exists, it is cached and provided as an output for the user. For more information about SES and its equivalence-discovering mechanism see Lagani et al. (2017).

The cornerstone upon which SES is built is the test of conditional independence used for computing $p_{XT|\mathbf{Z}}$. This holds as well for any other constraint-based feature selection algorithm (Aliferis et al., 2010). The test of conditional independence must be able to correctly deal with the idiosyncrasies of the data at hand. In case of temporal data, we propose to use the most appropriate test among the ones defined by Equations (4)-(7) in the main text and based on GLMMs and GEE models.

Algorithm 1 SES

```
Input:
Data set on n predictive variables V,
Target variable T,
Max conditioning set k, Significance threshold a

Output:
A set E of size n of variables sets Q_i, i = 1, ..., n
such that one can construct
a signature by selecting one and only one variable from each set Q_i
//Remaining variables
R ← V
//Currently selected variables
S ← ∅
//Sets of equivalences
Q_i ← i , for i = 1, ..., n

while R ≠ ∅ do
    for all X∈{R ∪ S} do
        if ∃Z ⊆ S \ {X}, |Z| ≤ k, s.t., p_{XT.Z} > a then
            R ← R \ {X}
            S ← S \ {X}

            //Identify statistical equivalences,
            //i.e., X and Y seem interchangeable
            if ∃Y∈Z, s.t., Z' ← (Z ∪ {X}) \ {Y}, p_{YT.Z'} > a then
                Q_Y ← Q_Y ∪ Q_X

            end if
        end if
    end for

    M = argmax_{X∈R} min_{Z⊆S,|Z|≤k} p_{XT.Z}
    R ← R \ {M}
    S ← S ∪ {M}

end while

Repeat the for-loop one last time
//Pack all the identified equivalences in one data structure
E ← ∅
for all i∈S do
    E ← E ∪ {Q_i}

end for

return E
```

3 Information about the datasets used in the experimentation

Tables S5 and S6 summarize some information about the datasets; namely the number of variables of subjects, relative group allocation (for the **Static-longitudinal** and **Static-distinct** scenarios only) and number of time points. Even though The numbers of variables are not really high enough, they are frequently met in bioinformatical applications. The sample sizes though are small and this is because wet lab experiments, especially with mice, and also experiments with human subjects can be quite expensive.

Table S5: Information about the datasets

Temporal-longitudinal scenario

Dataset	Number of Variables	Number of subjects	Number of time points
GDS5088	33295	11	4
GDS4395	54667	10	7
GDS4822	45037	9	5
GDS3326	54674	15	4
GDS3181	22283	12	3
GDS4258	54674	11	3
GDS3915	15921	9	5
GDS3432	40067	5	4

Temporal-distinct scenario

Dataset	Number of variables	No of subjects	Number of time points
GDS3859	45100	23	4
GDS972	15922	44	11
GDS947	16927	46	8
GDS964	15922	53	15
GDS2688	15923	45	11
GDS2135	22690	23	5

4 Supplementary methods

4.1 Assessing the equivalence of SES multiple signatures

For every fold of the cross validation we calculated the predicted values and thus the performance, of each signature as produced by SES. The mean of the standard deviation, minimum, maximum and coefficient of variation of all performances were then computed and are presented in Tables S8 and S10. The relevant boxplots of the performances appear in Figures S4 and S5.

Table S6: Information about the datasets

Static-longitudinal scenario

Dataset	Number of variables	No of subjects	Relative group allocation	Number of time points
GDS4146	22645	25	(0.64, 0.36)	5
GDS4518	24128	12	(0.50, 0.50)	3
GDS4820	54675	8	(0.50, 0.50)	3
GDS1840	15923	8	(0.50, 0.50)	4

Static-distinct scenario

Dataset	Number of variables	No of subjects	Relative group allocation	Number of time points
GDS4319	35556	112	(0.35, 0.32, 0.33)	6
GDS3924	38535	48	(0.25, 0.25, 0.50)	2
GDS3184	22575	47	(0.49, 0.51)	4
GDS3145	22690	64	(0.50, 0.50)	4
GDS3944	18116	32	(0.50, 0.50)	4
GDS2882	45101	40	(0.50, 0.50)	4
GDS2851	8799	23	(0.44, 0.56)	6
GDS1784	22690	36	(0.50, 0.50)	5
GDS2456	45101	39	(0.51, 0.49)	4

4.2 Using LASSO and gLASSO for the Temporal-disctinct and Static-distinct scenarios

Yang and Zou (2015) suggested the gLASSO for when there are categorical variables. In multinomial regression a continuous variable has $D - 1$ coefficients, where D denotes the number of values of the response variable. In the univariate regression, a categorical predictor variable has $d - 1$ coefficients, one for each of the $d - 1$ dummy variables, where d is the number of levels of this variable. The classical LASSO would penalise the coefficients ignoring this information. gLASSO overcomes this problem by shrinking towards zero, if necessary, all coefficients of a predictor variable simultaneously. This way, the variable is either included in the model or not, unlike LASSO, where a non-significant variable can stay in the model only because some of its coefficients are not zero.

5 Supplementary Results

5.1 glmmLasso scalability in high-dimensional data

Figure S1 shows how glmmLasso, SESglmm and SESgee (**Temporal-longitudinal** scenario) compare in terms of computational time. Both of the algorithms are written in R so the comparison is fair. When there are 2500 predictor variables, the time required by glmmLasso is 6

times the time required by SESglmm and SESgee. As seen from this Figure, the ratio of time increases as the number of variables increase.

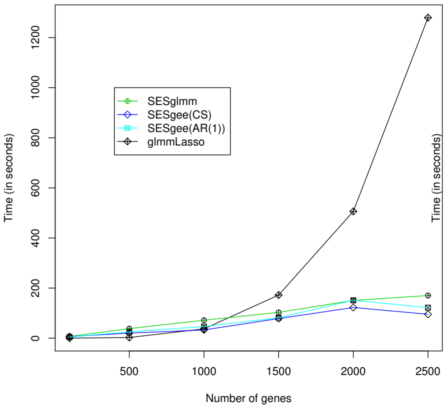

(a) GDS5088 Dataset

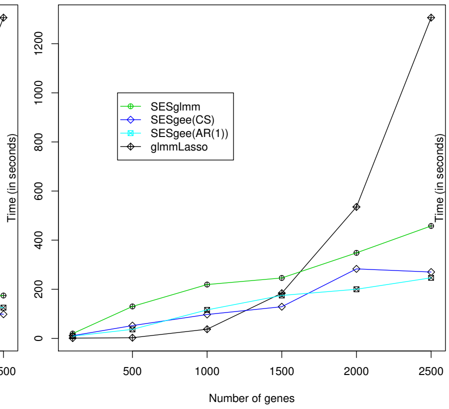

(b) GDS4395 Dataset

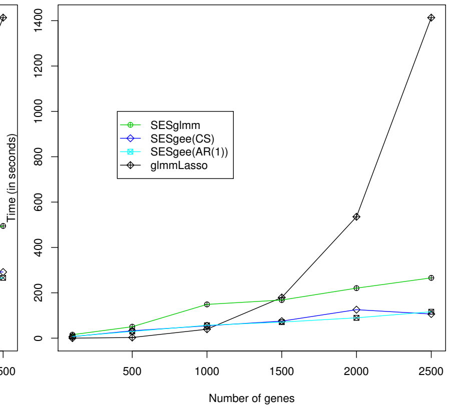

(c) GDS4822 Dataset

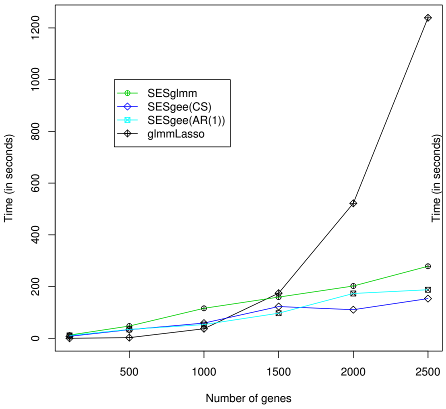

(d) GDS3326 Dataset

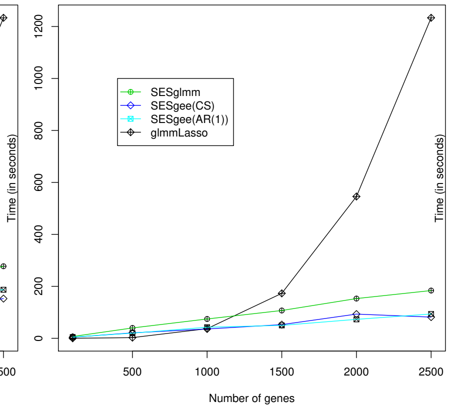

(e) GDS3181 Dataset

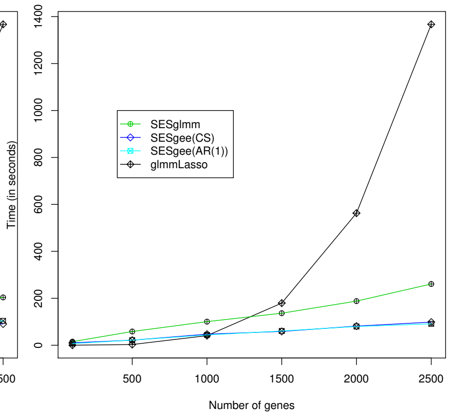

(f) GDS4258 Dataset

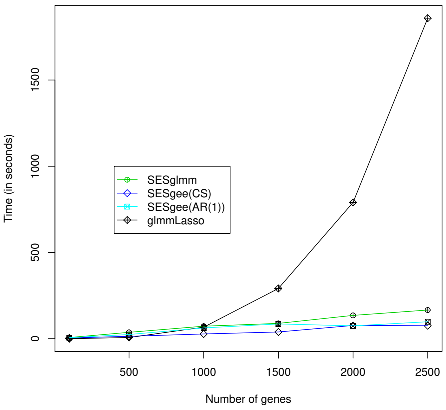

(g) GDS3915 Dataset

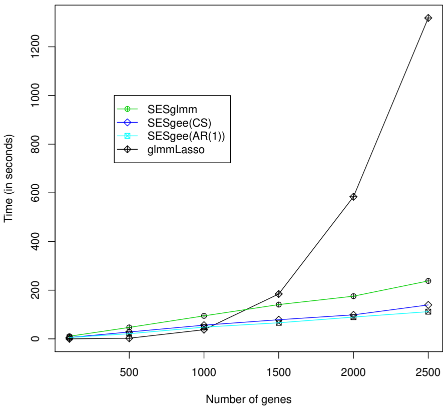

(h) GDS3432 Dataset

Figure S1: **Temporal-longitudinal** scenario: Time in seconds required by each of the three algorithms. Note, that for glmmLasso, the combined algorithm of gradient ascent and Fisher scoring was used. If no Fisher scoring was involved, the computational cost would be more than double of whhat currently is.

5.2 Computational requirements for SESglmm and SESgee and number of variables

Table 2 in the main text presents the computational cost, expressed in seconds, associated with SESglmm and SESgee. We detected a significant relationship between these two. At first, we removed the dataset GDS4395 as it was the most computationally expensive and would influence the results. For each method, (SESglmm, SESgee(CS), SESgee(AR(1))) there was a strong and significant relationship between the time and the number of variables. The degree of relationship increased when the logarithm for both measurements was applied, exhibiting a linear relationship between the two. We highlight that a quadratic term was not significant.

We then fitted a linear regression model combining all (logged) measurements, including two dummy variables indicating the method. Figure S3 shows graphically the results.

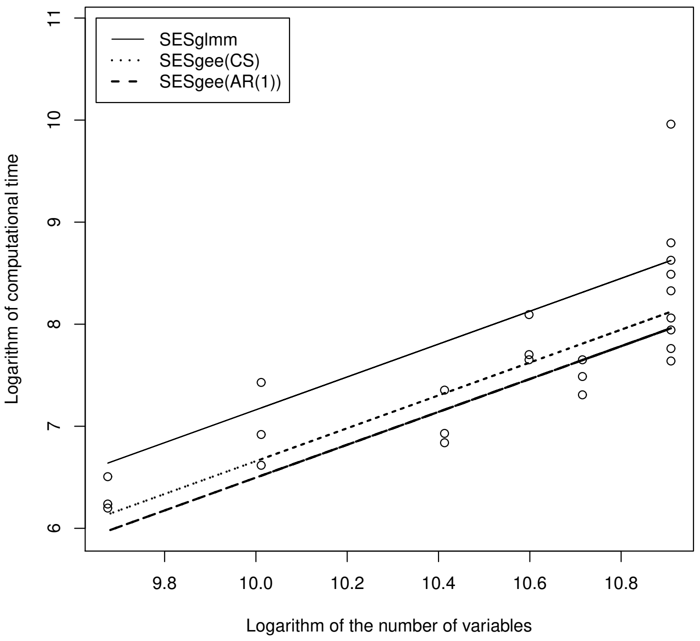

Figure S2: **Temporal-longitudinal** scenario: Logarithm of the number of variables versus the logarithm of the computational time for SESglmm and SESgee.

5.3 GDS5088: Comparison between SESglmm and glmmLasso

Figure S3 presents the box-plot of the MSPE between SESglmm and glmmLasso not shown in Figure 1(d) in the main text.

5.4 SES produces signatures with equivalent predictive performances

Figure S4 shows the box plots of the performances of all signatures as produced by the cross validations for the **Temporal-longitudinal** scenario. The performances are data dependent, as neither GLMM or GEE produces consistently better performances. The same is true for the variation in the performances. Figure S5 shows the performances of SES for the **Temporal-distinct** and **Static-distinct** scenarios. The same image as before is apparent here. For some

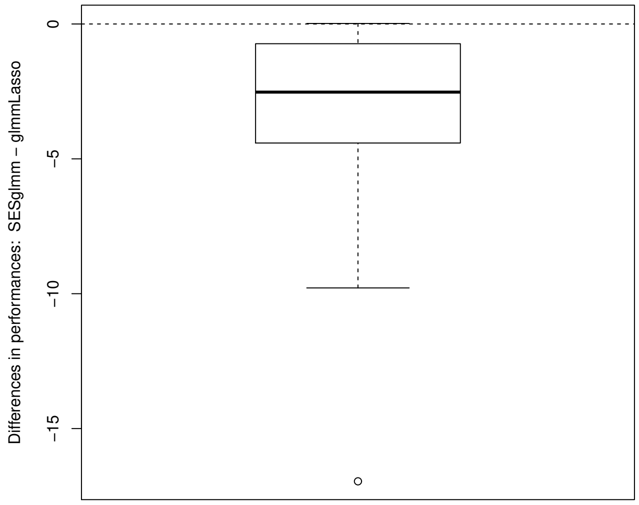

Figure S3: **Temporal-longitudinal** scenario: Dataset GDS5088. Difference in MSPE between SESglmm and glmmLasso (SESglmm-glmmLasso). Negative values indicate better performance of SESglmm.

datasets, there is little variation in the performances, whereas for others the performances vary greatly.

Let us remind ourselves that SES identifies equivalent variables, which are assumed to lead to statistically equivalent signatures. Having said that, it is natural to expect some discrepancies. If for example, the hypothesis is true, that the performance is uniform for all signatures, some signatures are expected to fall far from the others.

Table S7: **Temporal-longitudinal** scenario: Mean number of selected variables and signatures (the standard deviation appears inside the parentheses) produced by SESglmm and SESgees based on the the m-fold cross-validations.

	Mean selected variables			Mean number of signatures		
Dataset	SESglmm	SESgee(CS)	SESgee(AR(1))	SESglmm	SESgee(CS)	SESgee(AR(1))
GDS5088	4.96(0.95)	4.75(1.19)	5.12(1.26)	11.54(15.68)	113.50(169.18)	1188.17(2249.46)
GDS4395	6.27(0.69)	6.03(1.30)	5.73(1.36)	1.47(0.94)	86.42(151.41)	177.83(514.10)
GDS4822	5.33(0.87)	3.88(0.74)	5.00(0.72)	246.69(246.815)	400.96(686.61)	19.88(34.78)
GDS3326	6.21(0.83)	5.75(0.85)	6.29(0.81)	55.88(81.42)	2669.17(8237.89)	34.67(40.75)
GDS3181	4.25(0.68)	3.62(0.88)	4.21(0.59)	175.29(537.62)	254.79(531.30)	154.38(392.16)
GDS4258	4.67(0.49)	3.78(0.88)	4.17(0.86)	5.17(4.95)	487.61(540.92)	1765.44(3470.62)
GDS3432	3.88(0.80)	3.33(0.70)	4.33(0.64)	60.62(100.93)	1304.47(1996.69)	16584.79(30928.47)
GDS3915	5.92(0.88)	4.96(0.95)	5.83(0.96)	24.79(41.28)	816.50(1456.31)	8.62(13.32)

Figure S5 contain the box-plots of the performances of al signatures produced by SES via the m-fold cross validations for the **Temporal-distinct** and **Static-distinct** scenarios. More in-

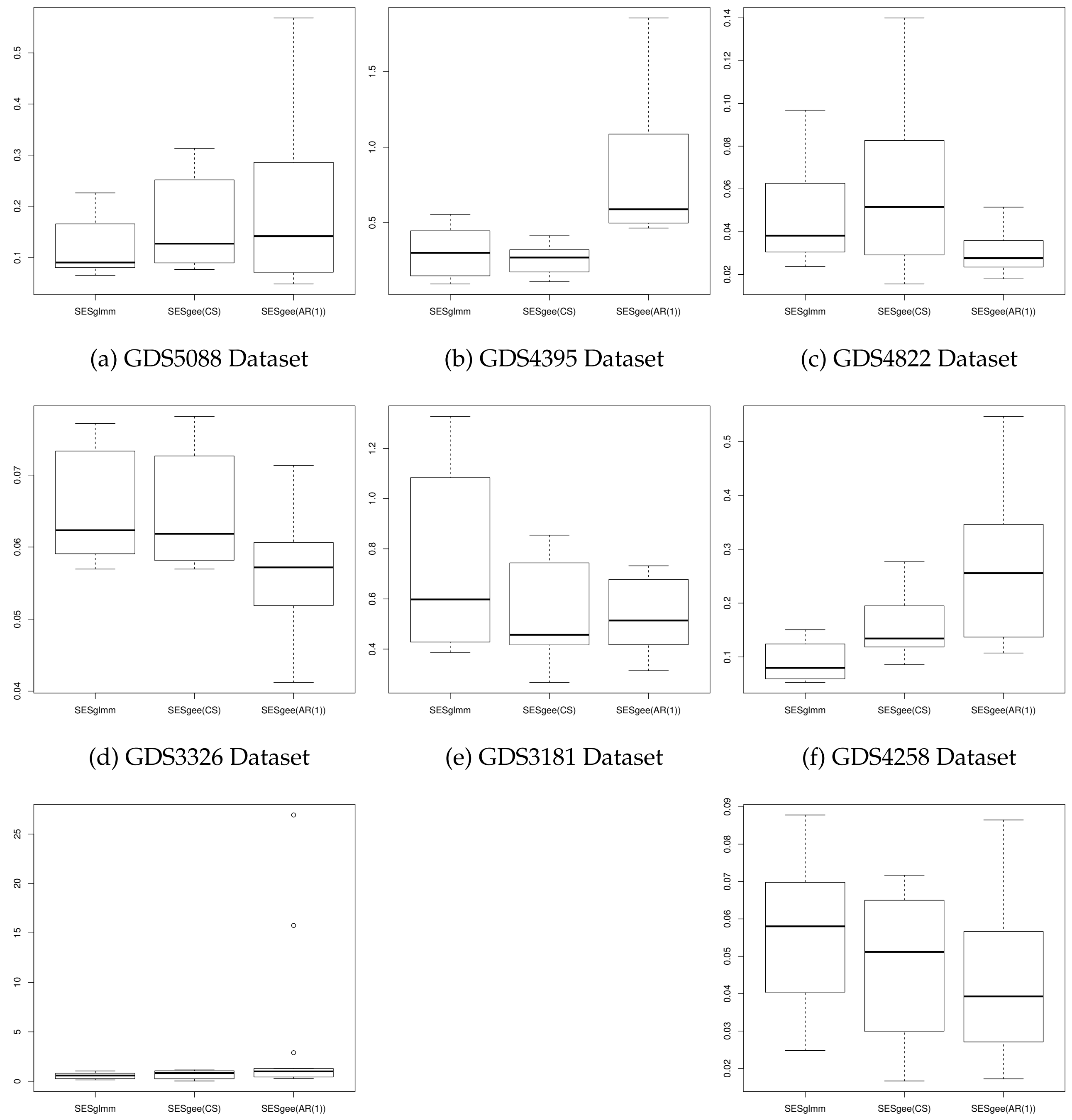

(a) GDS5088 Dataset (b) GDS4395 Dataset (c) GDS4822 Dataset

(d) GDS3326 Dataset (e) GDS3181 Dataset (f) GDS4258 Dataset

(g) GDS3915 Dataset (h) GDS3432 Dataset

Figure S4: **Temporal-longitudinal** scenario: Box plots of the performance of the signatures of the three SES methods as produced by the $6m$ cross validations.

formation characterizing these performances is available in Table S10.

The overall performances of SES and all types of LASSO under each Scenario were compared via a paired t-test was calculated whose p-value was computed using 9999 permutations. As expected, for Scenarion 1(a), SESglmm and SESgee do not produce statistically significant differences, whereas glmmLasso does differ. LASSO outperformed SES in the **Temporal-longitudinal** scenario, but this was something to be expected. When it comes to the **Static-**

Table S8: **Temporal-longitudinal** scenario: Means of the standard deviations, minimum, maximum and coefficient of variations of the the performance (MSPE) of all signatures, as produced by SESglmm and SESgees based on the m-fold cross-validations (every fold contains 6 pairs of the hyper-parameters a and k, thus $6m$ runs of SES each with at least one signature).

	Mean standard deviation			Mean coefficient of variation		
Dataset	SESglmm	SESgee(CS)	SESgee(AR(1))	SESglmm	SESgee(CS)	SESgee(AR(1))
GDS5088	0.017	0.163	0.246	0.163	0.671	0.868
GDS4395	0.048	1.22	0.185	0.144	0.224	0.237
GDS4822	0.020	0.022	0.003	0.396	0.356	0.109
GDS3326	0.008	0.008	0.005	0.115	0.116	0.094
GDS3181	0.254	0.115	0.135	0.310	0.202	0.230
GDS4258	0.015	0.058	0.091	0.143	0.355	0.328
GDS3432	0.171	1.033	2.367	0.307	2.483	13.590
GDS3915	0.007	0.017	0.006	0.152	0.470	0.125

	Mean minimum & maximum values		
	SESglmm	SESgee(CS)	SESgee(AR(1))
Dataset	(Min, Max)	(Min, Max)	(Min, Max)
GDS5088	(0.104, 0.140)	(0.091, 0.795)	(0.071, 1.175)
GDS4395	(0.279, 0.328)	(0.216, 0.350)	(0.652, 1.428)
GDS4822	(0.014, 0.132)	(0.019, 0.246)	(0.027, 0.034)
GDS3326	(0.056, 0.077)	(0.055, 0.077)	(0.049, 0.066)
GDS3181	(0.500, 1.232)	(0.450, 0.740)	(0.344, 0.864)
GDS4258	(0.073, 0.105)	(0.093, 0.314)	(0.097, 0.649)
GDS3432	(0.333, 1.004)	(0.165, 3.890)	(0.104, 8.18)
GDS3915	(0.046, 0.066)	(0.027, 0.093)	(0.037, 0.050)

Table S9: Scenario 1(a): Range of values of λ used in glmmLasso.

Dataset	GDS5088	GDS4395	GDS4822	GDS3326	GDS3181	GDS4258	GDS3432	GDS3915
Range of λ	[10-20]	[20-40]	[10-30]	[15-30]	[25-40]	[45-55]	[25-45]	[5-15]

longitudinal and **Static-distinct** scenarios, even though SES produced better results than LASSO the difference between the two seems not to be statistically significant.

6 Enrichment analysis on selected datasets

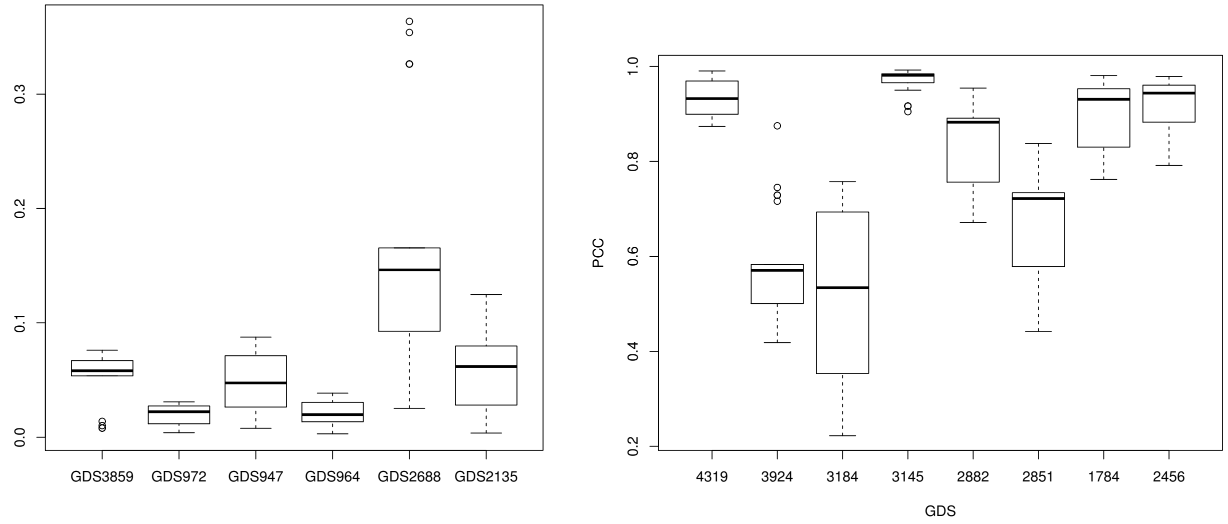

Figure S5: **Temporal-distinct** scenario: Box plots of the performance of the multiple signatures of SES as produced by the 6*m* cross validations (left) and **Static-distinct** scenario: Box plots of the performance of the multiple signatures of SES as produced by the 6*m* cross validations (right).

References

Aliferis, C. F., Statnikov, A. R., Tsamardinos, I., Mani, S., and Koutsoukos, X. D. (2010). Local Causal and Markov Blanket Induction for Causal Discovery and Feature Selection for Classification Part I : Algorithms and Empirical Evaluation. *Journal of Machine Learning Research*, 11:171–234.

Lagani, V., Athineou, G., Farcomeni, A., Tsagris, M., and Tsamardinos, I. (2017). Feature selection with the r package mxm: Discovering statistically-equivalent feature subsets. *Journal of Statistical Software*, 80.

Yang, Y. and Zou, H. (2015). A fast unified algorithm for solving group-lasso penalize learning problems. *Statistics and Computing*, 25(6):1129–1141.

Table S10: Means of the standard deviations, minimum, maximum and coefficient of variation (CV) of the the performance (MSPE) of all signatures, as produced by SEStimereg based on the m-fold cross-validations (every fold contains 6 pairs of the hyper-parameters a and k, thus $6m$ runs of SES each with at least one signature) for the **Temporal-distinct** and **Static-distinct** scenarios.

**Temporal-distinct scenario**			
Dataset	Standard deviation	CV	(Min, Max)
GDS3859	0.019	0.382	(0.019, 0.027)
GDS972	0.002	0.158	(0.018, 0.022)
GDS947	0.001	0.192	(0.038, 0.058)
GDS964	0.003	0.140	(0.018, 0.025)
GDS2688	0.019	0.157	(0.136, 0.206)
GDS2135	0.037	0.499	(0.019, 0.158)
**Static-distinct scenario**			
Dataset	Standard deviation	CV	(Min, Max)
GDS4319	0.004	0.043	(0.775, 0.994)
GDS3924	0.165	0.300	(0.257, 0.875)
GDS3184	0.139	0.308	(0.213, 0.796)
GDS3145	0.052	0.054	(0.779, 1.000)
GDS3944	–	–	(–, –)
GDS2882	0.118	0.145	(0.554, 1.000)
GDS2851	0.152	0.228	(0.366, 0.861)
GDS1784	0.126	0.110	(0.412, 1.000)
GDS2456	0.116	0.132	(0.291, 1.000)

Table S11: Permutation based (9999 permutations) p-values using the paired t-test statistic as the test statistic for comparing the overall performances across the different methods and Scenarios.

**Temporal-longitudinal scenario**	
Methods	p-value
SESglmm Vs SESgee(CS)	0.7265
SESglmm Vs SESgee(AR(1))	0.6716
SESgee(CS) Vs SESgee(AR(1))	0.3706
SESglmm Vs glmmLasso	0.0158
SESgee(CS) Vs glmmLasso	0.0001
SESgee(AR(1)) Vs glmmLasso	0.0001
**Temporal-static scenario**	
Methods	p-value
SES Vs LASSO	0.0001
**Static-longitudinal scenario**	
Methods	p-value
SES Vs GLASSO	0.4024
**Static-distinct scenario**	
Methods	p-value
SES Vs LASSO	0.068

Table S12: KEGG Pathways significantly enriched at FDR level of 0.1 for the SES signature selected on Dataset GDS947.

ID	Description	p-value	adjusted p-value
mmu04978	Mineral absorption	0.017	0.056
mmu01524	Platinum drug resistance	0.028	0.056
mmu03010	Ribosome	0.064	0.086
mmu04010	MAPK signaling pathway	0.091	0.091

Table S13: KEGG Pathways significantly enriched at FDR level of 0.1 for the SES signature selected on Dataset GDS972.

ID	Description	p-value	adjusted p-value
rno05134	Legionellosis	0.021	0.07
rno04540	Gap junction	0.031	0.07
rno05162	Measles	0.048	0.07
rno04141	Protein processing in endoplasmic reticulum	0.058	0.07
rno05164	Influenza A	0.06	0.07
rno04145	Phagosome	0.07	0.07

Table S14: KEGG Pathways significantly enriched at FDR level of 0.1 for the SES signature selected on Dataset GDS2135.

ID	Description	p-value	adjusted p-value
mmu04512	ECM-receptor interaction	0.000	0.004
mmu05222	Small cell lung cancer	0.000	0.004
mmu04510	Focal adhesion	0.002	0.015
mmu04151	PI3K-Akt signaling pathway	0.005	0.033
mmu05200	Pathways in cancer	0.007	0.034
mmu05140	Leishmaniasis	0.025	0.062
mmu05412	Arrhythmogenic right ventricular cardiomyopathy (ARVC)	0.026	0.062
mmu05133	Pertussis	0.028	0.062
mmu05100	Bacterial invasion of epithelial cells	0.028	0.062
mmu05410	Hypertrophic cardiomyopathy (HCM)	0.031	0.062
mmu05414	Dilated cardiomyopathy	0.033	0.062
mmu04974	Protein digestion and absorption	0.033	0.062
mmu03015	mRNA surveillance pathway	0.035	0.062
mmu04933	AGE-RAGE signaling pathway in diabetic complications	0.037	0.062
mmu05146	Amoebiasis	0.039	0.062
mmu05145	Toxoplasmosis	0.04	0.062
mmu04670	Leukocyte transendothelial migration	0.042	0.062
mmu04611	Platelet activation	0.045	0.063
mmu04530	Tight junction	0.061	0.075
mmu04514	Cell adhesion molecules (CAMs)	0.061	0.075
mmu04360	Axon guidance	0.064	0.075
mmu04145	Phagosome	0.066	0.075
mmu05205	Proteoglycans in cancer	0.075	0.077
mmu04015	Rap1 signaling pathway	0.077	0.077
mmu04810	Regulation of actin cytoskeleton	0.077	0.077

Table S15: KEGG Pathways significantly enriched at FDR level of 0.1 for the SES signature selected on Dataset GDS2456.

ID	Description	p-value	adjusted p-value
mmu00260	Glycine, serine and threonine metabolism	0.015	0.042
mmu03050	Proteasome	0.017	0.042
mmu01230	Biosynthesis of amino acids	0.029	0.048
mmu01200	Carbon metabolism	0.043	0.054
mmu04141	Protein processing in endoplasmic reticulum	0.061	0.061

Table S16: KEGG Pathways significantly enriched at FDR level of 0.1 for the SES signature selected on Dataset GDS2688.

ID	Description	p-value	adjusted p-value
rno04962	Vasopressin-regulated water reabsorption	0.021	0.066
rno04721	Synaptic vesicle cycle	0.030	0.066
rno05100	Bacterial invasion of epithelial cells	0.038	0.066
rno04512	ECM-receptor interaction	0.040	0.066
rno04727	GABAergic synapse	0.043	0.066
rno04974	Protein digestion and absorption	0.044	0.066

Table S17: KEGG Pathways significantly enriched at FDR level of 0.1 for the SES signature selected on Dataset GDS2882.

ID	Description	p-value	adjusted p-value
mmu05222	Small cell lung cancer	0.001	0.007
mmu04657	IL-17 signaling pathway	0.001	0.007
mmu04064	NF-kappa B signaling pathway	0.001	0.007
mmu04668	TNF signaling pathway	0.001	0.007
mmu05167	Kaposi's sarcoma-associated herpesvirus infection	0.004	0.018
mmu05203	Viral carcinogenesis	0.005	0.020
mmu05200	Pathways in cancer	0.013	0.047
mmu04923	Regulation of lipolysis in adipocytes	0.027	0.069
mmu04370	VEGF signaling pathway	0.028	0.069
mmu04913	Ovarian steroidogenesis	0.028	0.069
mmu05140	Leishmaniasis	0.033	0.069
mmu04622	RIG-I-like receptor signaling pathway	0.033	0.069
mmu00590	Arachidonic acid metabolism	0.043	0.080
mmu05204	Chemical carcinogenesis	0.045	0.080
mmu04620	Toll-like receptor signaling pathway	0.048	0.080
mmu04919	Thyroid hormone signaling pathway	0.056	0.087
mmu04726	Serotonergic synapse	0.064	0.088
mmu05160	Hepatitis C	0.065	0.088
mmu05161	Hepatitis B	0.069	0.088
mmu04723	Retrograde endocannabinoid signaling	0.072	0.088
mmu04921	Oxytocin signaling pathway	0.074	0.088
mmu04621	NOD-like receptor signaling pathway	0.081	0.092

Table S18: KEGG Pathways significantly enriched at FDR level of 0.1 for the SES signature selected on Dataset GDS3145.

ID	Description	p-value	adjusted p-value
mmu03030	DNA replication	0.013	0.076
mmu00561	Glycerolipid metabolism	0.022	0.076
mmu01521	EGFR tyrosine kinase inhibitor resistance	0.029	0.076
mmu03320	PPAR signaling pathway	0.031	0.076
mmu04066	HIF-1 signaling pathway	0.038	0.076
mmu04110	Cell cycle	0.045	0.076
mmu04371	Apelin signaling pathway	0.051	0.076
mmu04910	Insulin signaling pathway	0.051	0.076
mmu04150	mTOR signaling pathway	0.056	0.076
mmu05010	Alzheimer′s disease	0.064	0.076
mmu03010	Ribosome	0.064	0.076
mmu05205	Proteoglycans in cancer	0.075	0.081

Table S19: KEGG Pathways significantly enriched at FDR level of 0.1 for the SES signature selected on Dataset GDS3326.

ID	Description	p-value	adjusted p-value
hsa00512	Mucin type O-glycan biosynthesis	0.009	0.034
hsa05323	Rheumatoid arthritis	0.025	0.035
hsa04064	NF-kappa B signaling pathway	0.026	0.035
hsa04060	Cytokine-cytokine receptor interaction 0.073 0.073		

Table S20: KEGG Pathways significantly enriched at FDR level of 0.1 for the SES signature selected on Dataset GDS3915.

ID	Description	p-value	adjusted p-value
rno04964	Proximal tubule bicarbonate reclamation	0.013	0.077
rno00592	alpha-Linolenic acid metabolism	0.015	0.077
rno04960	Aldosterone-regulated sodium reabsorption	0.023	0.077
rno00591	Linoleic acid metabolism	0.025	0.077
rno04973	Carbohydrate digestion and absorption	0.025	0.077
rno00565	Ether lipid metabolism	0.027	0.077
rno04978	Mineral absorption	0.027	0.077
rno04961	Endocrine and other factor-regulated calcium reabsorption	0.030	0.077
rno04923	Regulation of lipolysis in adipocytes	0.034	0.077
rno04918	Thyroid hormone synthesis	0.043	0.077
rno04976	Bile secretion	0.043	0.077
rno04971	Gastric acid secretion	0.044	0.077
rno04970	Salivary secretion	0.046	0.077
rno05100	Bacterial invasion of epithelial cells	0.047	0.077
rno04260	Cardiac muscle contraction	0.048	0.077
rno00590	Arachidonic acid metabolism	0.049	0.077
rno04911	Insulin secretion	0.051	0.077
rno04666	Fc gamma R-mediated phagocytosis	0.052	0.077
rno04974	Protein digestion and absorption	0.055	0.077
rno00564	Glycerophospholipid metabolism	0.058	0.077
rno04972	Pancreatic secretion	0.058	0.077
rno04713	Circadian entrainment	0.058	0.077
rno04919	Thyroid hormone signaling pathway	0.069	0.087

Table S21: KEGG Pathways significantly enriched at FDR level of 0.1 for the SES signature selected on Dataset GDS4258.

ID	Description	p-value	adjusted p-value
hsa04710	Circadian rhythm	0.013	0.076
hsa04012	ErbB signaling pathway	0.035	0.089
hsa04390	Hippo signaling pathway	0.062	0.089
hsa05202	Transcriptional misregulation in cancer	0.072	0.089
hsa04062	Chemokine signaling pathway	0.074	0.089

Table S22: KEGG Pathways significantly enriched at FDR level of 0.1 for the SES signature selected on Dataset GDS4395.

ID	Description	p-value	adjusted p-value
hsa04137	Mitophagy - animal	0.027	0.099
hsa04924	Renin secretion	0.027	0.099
hsa04270	Vascular smooth muscle contraction	0.049	0.099
hsa05012	Parkinson's disease	0.057	0.099
hsa04022	cGMP-PKG signaling pathway	0.066	0.099
hsa05010	Alzheimer's disease	0.069	0.099
hsa04020	Calcium signaling pathway	0.073	0.099
hsa04024	cAMP signaling pathway	0.08	0.099

Table S23: KEGG Pathways significantly enriched at FDR level of 0.1 for the SES signature selected on Dataset GDS4822.

ID	Description	p-value	adjusted p-value
mmu03018	RNA degradation	0.02	0.042
mmu04925	Aldosterone synthesis and secretion	0.021	0.042
mmu04010	MAPK signaling pathway	0.061	0.082
mmu04151	PI3K-Akt signaling pathway	0.084	0.084
